# DNAshape: a method for the high-throughput prediction of DNA structural features on a genomic scale

**DOI:** 10.1093/nar/gkt437

**Published:** 2013-05-22

**Authors:** Tianyin Zhou, Lin Yang, Yan Lu, Iris Dror, Ana Carolina Dantas Machado, Tahereh Ghane, Rosa Di Felice, Remo Rohs

**Affiliations:** ^1^Molecular and Computational Biology Program, Department of Biological Sciences, ^2^Department of Physics and Astronomy, ^3^Department of Chemistry, ^4^Department of Computer Science and ^5^Norris Comprehensive Cancer Center, University of Southern California, Los Angeles, CA 90089, USA

## Abstract

We present a method and web server for predicting DNA structural features in a high-throughput (HT) manner for massive sequence data. This approach provides the framework for the integration of DNA sequence and shape analyses in genome-wide studies. The HT methodology uses a sliding-window approach to mine DNA structural information obtained from Monte Carlo simulations. It requires only nucleotide sequence as input and instantly predicts multiple structural features of DNA (minor groove width, roll, propeller twist and helix twist). The results of rigorous validations of the HT predictions based on DNA structures solved by X-ray crystallography and NMR spectroscopy, hydroxyl radical cleavage data, statistical analysis and cross-validation, and molecular dynamics simulations provide strong confidence in this approach. The DNAshape web server is freely available at http://rohslab.cmb.usc.edu/DNAshape/.

## INTRODUCTION

An increasing number of structural biology and genomics studies associate protein–DNA binding with the recognition of the three-dimensional DNA structure, or ‘DNA shape’ (1). DNA shape readout (2) plays an important role in determining the DNA binding preferences of transcription factors (3,4) and other DNA binding proteins (5–8). Whereas DNA shape is sequence dependent, degeneracy in the sequence–structure relationship enables the formation of very similar shapes by dissimilar sequences or, in turn, dramatic effects on structure in an extended region by only a single-nucleotide substitution (9).

In early studies, all of the available crystal structures of DNA fragments and protein–DNA complexes were analysed to derive the average conformations for the 10 unique dinucleotides (10). Based on the many more high-resolution structures that have been solved and analysed in recent years, it is now apparent that longer DNA segments must be characterized to capture the sequence–structure degeneracy of DNA (1). Such structural information, which can be retrieved from X-ray crystallography or NMR spectroscopy data, ideally provides information on the three-dimensional structure of a DNA binding site prior to and after protein binding. However, such data are unavailable for most sequences for their unbound or ‘naked’ states (11). Consequently, molecular simulations are the only available approach to deduce atomic information on intrinsic DNA structure.

Recent efforts to characterize the structures of all 136 unique tetranucleotides have used all-atom molecular dynamics (MD) simulations of either 136 dodecamers (12) or 39 duplexes of 18 base pairs (bp) in length (13). However, in both designs most tetranucleotides occur only in the context of a single sequence, which limits the ability for a statistically robust comparison of the simulation results with experimental data. Due to their relatively time-consuming nature, simulation methods make structural analysis of massive DNA sequence data unfeasible (see Supplementary Materials and Methods).

We previously improved the efficiency of conformational sampling by reducing the number of degrees of freedom in the system. Our Monte Carlo (MC) approach uses collective and internal variables for each nucleotide (14,15), an analytic chain closure with associated Jacobians (16) and an implicit solvent description (17) to realize improved sampling efficiency. This approach has enabled us to study a large number of DNA fragments. Although we successfully applied our MC approach in various studies of protein–DNA recognition (3,7,11,18,19), to bring this to the genomic scale, we have recently developed the methodology for facilitating MC data in high-throughput (HT) studies of DNA shape (4,8,18).

Through the mining of atomic resolution data obtained from MC simulations (14–16) of numerous DNA fragments, our HT approach is designed to predict various important structural features of DNA for essentially any length or number of sequences. We applied a prior version of the underlying method to the minor groove width (MGW) prediction of Hox-binding sites derived from HT *in vitro* assays (18). Here, we present a method that improves the accuracy of this approach and expands the prediction to additional DNA structural features. Given their importance in DNA shape readout, the predicted features include MGW, Roll, propeller twist (ProT) and helix twist (HelT) (see Supplementary Figure S1 for schematic representations) (3,14,18). While we emphasize that protein–DNA recognition is a dynamic process, the predicted structural features reveal preferred conformations that are intrinsic to a given DNA sequence. We demonstrate the robustness of our method through extensive validation with massive experimental and computational data. Our HT method underlying the DNAshape web server can be used to predict DNA structural features of the entire yeast genome at nucleotide resolution in less than 1 min on a single processor.

## METHODOLOGY

**Figure 1.**
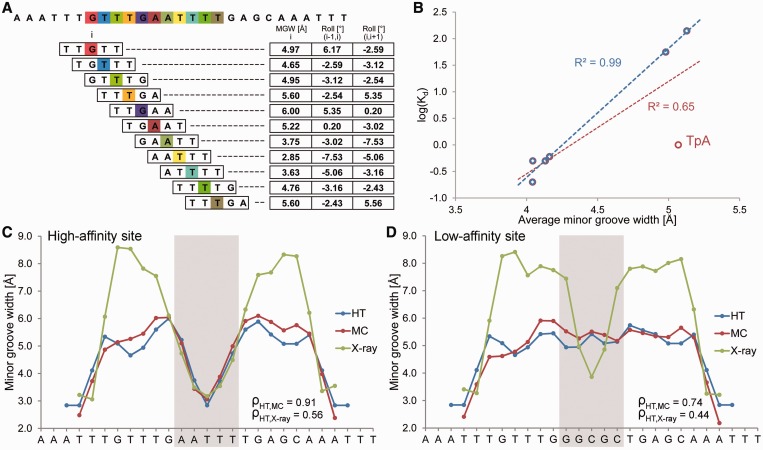
Pentamer model for HT prediction and validation of the HT approach using Fis-binding sites. (**A**) MC predictions were mined with a sliding-pentamer window, and a query table of average structural features characterizing either the central bp (e.g., MGW) or the two central bp steps (e.g., Roll) of a pentamer was assembled to predict the structural features of any length of DNA. (**B**) HT predictions of the average MGW over the five central bp of seven Fis-binding sites (6) correlate with the logarithm of binding affinity (red). The predictive power further increases for six sequences (blue) after one sequence with a TpA step is removed (F25; Supplementary Table S2). The MGW as a function of sequence is predicted for (**C**) high-affinity (*K_d_* = 0.2 nM) and (**D**) low-affinity (*K_d_* = 140 nM) binding sites using the HT (blue) and MC (red) approaches and compared with X-ray data (green) of protein–DNA complexes (PDB IDs in Supplementary Materials and Methods). The large positive MGW values observed in X-ray data are usually due to crystal packing and are not observed in solution. Spearman’s rank correlation coefficients (ρ) demonstrate the statistical similarity between the predicted and experimental MGW profiles.

DNA shape features at a single-nucleotide position are determined by the sequence context of the corresponding bp. The context can include the immediate neighbors of a bp or a larger number of adjacent bp. Given that pentamers account for the nearest and next-nearest neighbors of their central positions, a pentamer model is a reasonably simplified approach that takes sequence context into account. Thus, the structural features at each bp position can be characterized as a function of its pentameric environment. Assuming that the structure of each of the 512 unique pentamers is known, we can use a ‘sliding pentamer model’ to derive the structural features of DNA molecules of any length and with any sequence in an instant manner (Figure 1A).

To assemble the pentamer data, we generated a large training dataset of MC trajectories for 2121 different DNA fragments of 12–27 bp in length (see Supplementary Table S1 for list of sequences and Supplementary Materials and Methods for further details on MC method). Our MC data provide full coverage of all 512 unique pentamers, each occurring on average 44 times in the training dataset. The multiple occurrence of each pentamer in different sequence contexts is used to account for the effect of the flanking sequences through averaging.

For each MC prediction, the structural features at single-nucleotide resolution were analysed with Curves (20). We used a modified definition for assigning MGW measurements to a bp, in which the MGW definition is independent of the usage of strand 1 or 2 of the DNA duplex as the leading strand. Specifically, the MGW at a given bp was calculated by averaging groove width measurements over three levels [−1, 0, +1] surrounding the plane of a given bp, with levels 0 and +1 representing the first two levels calculated by Curves (20) for a nucleotide, and −1 being the last level of the preceding nucleotide. This definition includes 5′ and 3′ inter-bp values and defines MGW as a direction-independent measure.

Using a sliding-pentamer window, we decomposed each MC prediction into a set of overlapping pentamers (Figure 1A). All occurrences of a given pentamer in our MC dataset were collected and the average structural features were calculated for the central bp (MGW and ProT) or the two central bp steps (Roll and HelT). These average structural parameters for each pentamer were stored in a query table, with the pentamer sequence serving as the search key (Figure 1A). The data could then be used to predict DNA shape features of arbitrary sequences.

## WEB SERVER

The input is a nucleotide sequence ranging from 5 to 10^6^ bp in length. Multiple sequences can be entered, either one sequence per line or in FASTA format.

The output data are predicted values for MGW and ProT as a function of bp and for Roll and HelT as a function of bp steps (Supplementary Figure S1). Structural features are predicted for the entire sequence except for the two terminal bp or one bp step at each end. Predictions for the different structural features are organized in individual tabs. Our server provides actual values in text format along with plots visualizing the structural features of every analysed sequence as a function of the nucleotide sequence.

## VALIDATION

We compared DNA shape predictions for seven DNA binding sites that exhibit Fis-binding affinities differing by three orders of magnitude (6). The Fis protein binds various DNA sequences with the binding affinity depending on MGW in the central region of its binding site (6). The predicted MGW, averaged over the region of the five central nucleotides, correlates with the logarithm of binding affinity with *R^2^* = 0.65 (Figure 1B). When we excluded a particular sequence with a central TpA ‘hinge’ step (denoted F25; see Supplementary Table S2 for list of sequences) due to its high flexibility (1), the correlation is even stronger with *R^2^* = 0.99 (Figure 1B). This finding suggests a future application of DNA shape analysis in predicting binding affinity. For the two sequences with the highest and lowest binding affinities (6), the MGW predictions of the DNA targets show that the high-affinity site assumes a groove geometry in its unbound state similar to that observed in the complex (Figure 1C). In contrast, the low-affinity site must be deformed upon binding (Figure 1D).

**Figure 2.**
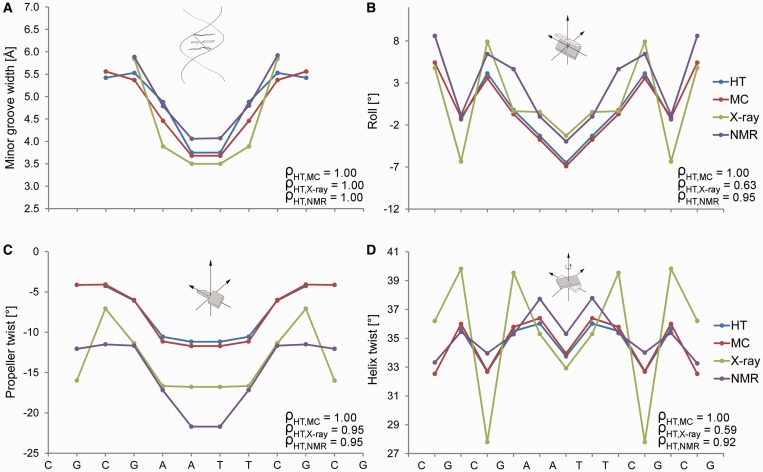
Validation of HT predictions using the Dickerson dodecamer. Structural features (**A**) MGW, (**B**) Roll, (**C**) ProT and (**D**) HelT of the palindromic Dickerson dodecamer are predicted with the HT (blue) and MC (red) approaches. These features are compared with the symmetrized average profiles derived from eight crystal structures (green) without chemical modifications and the average profiles derived from 10 NMR structures (purple) using RDC (PDB IDs in Supplementary Table S3). The more extreme HelT values observed in X-ray data are usually due to crystal packing and are not observed in solution. Spearman’s rank correlation coefficients (ρ) demonstrate the statistical similarity between the predicted and experimental structural feature profiles, which we symmetrized according to the palindromic sequence.

We explicitly demonstrate the performance of our predictions for the Dickerson dodecamer of the palindromic sequence CGCGAATTCGCG, which is the experimentally most extensively studied DNA molecule (11). Crystal-packing effects lead to an asymmetric X-ray structure of this molecule, which we can eliminate through symmetrization due to the palindromic symmetry of its sequence (14). The refinement of NMR structures requires numerous NOE constraints, which are sparse in DNA (11), but additional NMR data can be derived from residual dipolar coupling (RDC) (21). The sequence-dependent patterns of the MGW, Roll, ProT and HelT predictions obtained with our HT server agree very well with average measurements of shape features for 8 X-ray and 10 NMR structures (Figure 2; see Supplementary Table S3 for PDB IDs). We also compared the HT prediction of structural features for the Dickerson dodecamer with data from a 100-ns MD simulation (see Supplementary Materials and Methods for details on MD protocol). The HT and MC predictions are consistent with the MD data, particularly for the sequence-dependent patterns of all four predicted DNA shape parameters (Supplementary Figure S2). We recently also demonstrated the high correlation of the MC prediction for MGW of the Dickerson dodecamer with hydroxyl radical (OH) cleavage intensity measurements (19).

**Figure 3.**
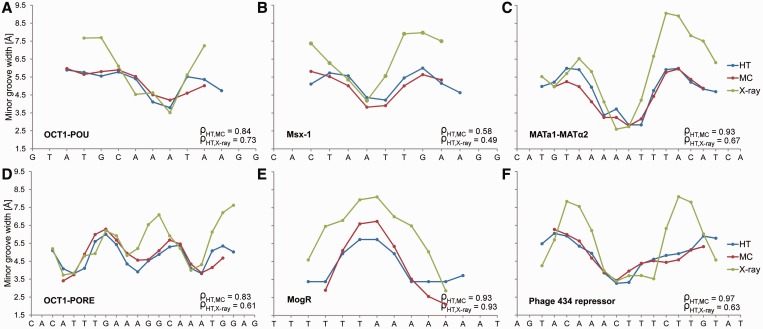
Validation of HT predictions using protein–DNA binding sites. (**A–F**) MGW for the DNA binding sites of six proteins, for which DNA shape readout was previously observed (1), are predicted using the HT (blue) and MC (red) approaches and compared with X-ray data (green) of protein–DNA complexes (PDB IDs in Supplementary Materials and Methods). The large positive MGW values observed in X-ray data are usually due to crystal packing and are not observed in solution. Therefore, qualitative MGW patterns (minima versus maxima) are the more essential characteristics compared to actual values. The MGW minima correlate with regions of enhanced negative electrostatic potential, thus providing binding sites for basic arginine side chains (1). Spearman’s rank correlation coefficients (ρ) demonstrate the statistical similarity between the predicted and experimental MGW profiles.

We next focused on the MGW of the DNA binding sites of six proteins for which we previously established the importance of minor groove shape readout (1). The HT predictions show very good agreement with MC predictions of the MGW profiles (Figure 3). Because our HT method essentially yields the average results of multiple MC predictions (thereby decreasing the impact of potential sampling artifacts), the HT predictions tend to lack extreme variations in structural features that are exhibited by MC or MD simulations (Supplementary Figure S2). We also validated our HT predictions with the crystal structures of protein–DNA complexes for these six examples (1). The MGW maxima observed in the crystal structures assume more extreme values due to crystal-packing effects compared to the 5.8 Å B-DNA average. Nevertheless, our HT method predicted the MGW patterns observed in the six crystal structures very well, as indicated by the Spearman’s rank correlation coefficients (range 0.49–0.93).

**Figure 4.**
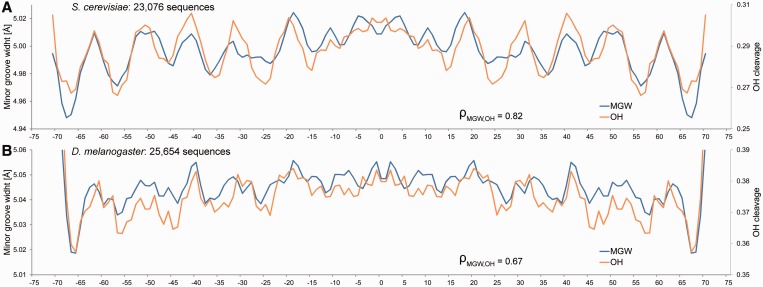
Validation of HT predictions using nucleosome binding sites. The MGW is predicted for (**A**) 23 076 yeast and (**B**) 25 654 fly *in vivo* nucleosome binding sites (22,23), and its average is compared with previously published OH-cleavage data derived from ORChID2 (19). HT predictions of MGW (blue) and OH-cleavage intensity (orange) are highly correlated, both revealing the 10-bp periodicity observed in dinucleotide propensity (1). Spearman’s rank correlation coefficients (ρ) demonstrate the statistical similarity between the predicted MGW and OH-cleavage intensity profiles.

OH-cleavage intensity is a qualitative measure for MGW in solution. OH-cleavage intensities, which originate from nucleotides on each strand that are closest across the minor groove, have been compiled in the ORChID2 server (19) through a sliding-window approach similar to the one described here. This server enables the prediction of OH-cleavage patterns for DNA duplexes of any length. ORChID2 data (19) were used to validate HT predictions of large numbers of sequences. We predicted the average MGW profiles of 23 076 yeast (22) and 25 654 fly (23) *in vivo* nucleosome binding sites, respectively. The HT predictions of MGW are highly consistent with the OH data for the same sequence datasets (Spearman’s rank correlation coefficients of 0.82 and 0.67, respectively) and exhibit a 10-bp periodicity (Figure 4), consistent with the results of known dinucleotide analyses (24,25), suggesting that DNA shape is recognized by histones (1).

In addition to the MGW comparison, we validated the HT prediction of ProT, Roll and HelT for five additional examples of protein–DNA binding sites (Supplementary Figures S3–S7). All four DNA shape parameters of these DNA targets are well predicted with our HT approach, according to Spearman’s rank correlation coefficients. Interestingly, the overestimation of MGW and underestimation of HelT that are generally reported for MD simulations (13,26,27) are not observed when our HT method is used.

We also performed the massive validation of our HT predictions using all available experimental DNA structures from the Protein Data Bank (PDB). We collected structures from previously characterized datasets (19) solved by X-ray crystallography (760 bound DNAs; 46 unbound DNAs) and NMR spectroscopy (90 unbound DNAs) as validation datasets (see Supplementary Table S3 for list of PDB IDs). Only structures with at least one helical turn and no chemical modifications were included. We organized the predicted and experimentally-derived shape parameters into separate ‘structural feature vectors’. The elements of each vector were ordered by the position of each bp (MGW and Roll) or bp step (ProT and HelT) to which they corresponded.

We evaluated the correlation between these vectors to obtain a quantitative measure of agreement between our HT predictions and the experimental structures. For this comparison to be meaningful, however, two problems needed to be addressed. First, for structures of short length and with minor structural variations, quantitative comparisons of the HT prediction with experimental measurements are vulnerable to small fluctuations. Second, some experimental structures exhibit drastic deformations, mainly due to crystal packing but in some cases also induced by protein binding. Such deformations yield unusual structural features particularly in end regions that are not observed in solution.

To address these issues, we identified regions of extreme deformations in experimental structures, which satisfied one of the following criteria: (i) MGW > 8.5 Å or < 1.5 Å (∼5.8 Å in B-DNA); (ii) HelT > 45° (∼35° in B-DNA); and (iii) |Roll| > 20° (∼0° in B-DNA). We removed the elements of the structural feature vectors corresponding to these deformed regions. The 3-bp flanks 5′ or 3′ of a deformed region were also removed from the validation dataset. For each of the structural features (MGW, Roll, ProT and HelT), we concatenated the structural feature vectors from all structures into one single vector. As a result of the concatenation, each structural feature vector can contain elements that represent up to 3000 bp, depending on the size of the validation dataset. We then calculated Spearman’s rank correlation coefficients between the structural feature vectors to obtain a quantitative measure of agreement between the HT predictions and experimental structures (Supplementary Table S4).

For the largest dataset of bound DNAs derived from X-ray data, we achieved Spearman’s rank correlation coefficients of 0.67 for MGW, 0.63 for Roll, 0.55 for ProT and 0.54 for HelT. Some features were less well predicted in unbound DNA structures, with the exception of the unbound Dickerson dodecamer that shows excellent agreement (Spearman’s rank correlation coefficients of 1.0 for MGW, 0.63 for Roll, 0.95 for ProT and 0.54 for HelT for X-ray data and 1.0 for MGW, 0.95 for Roll, 0.95 for ProT and 0.95 for HelT for NMR data; Supplementary Table S4). This observation is due to (i) the much smaller datasets of experimental structures for unbound DNAs versus protein-bound DNAs; (ii) the stronger crystal-packing deformations of unbound DNAs compared to DNAs in complexes; and (iii) the smaller number of available NMR-derived constraints for DNA (11). The latter two effects were not observed for the Dickerson dodecamer, and the accuracy of HT prediction for this particular form of unbound DNA is comparable and even superior to that for bound DNAs. This result can be explained by the removal of crystal-packing effects through symmetrization according to the palindromic sequence (14). With regard to the NMR structures of unbound DNAs, the Dickerson dodecamer is the only sequence for which RDC data were used in the structural refinement (21). The Spearman’s rank correlation coefficients for HelT between HT predictions and experimental data are lower for X-ray than for NMR data. This finding is likely due to the extreme values of HelT seen in crystal structures, which are not observed in solution-state NMR structures (Figure 2D).

We further investigated the HT prediction of Roll and HelT for the 10 unique dinucleotides. The dinucleotide-specific pattern of these helical parameters is well captured by HT predictions compared to X-ray data (Supplementary Figure S8). In particular, the MC-based HT method accurately predicts the average HelT over all dinucleotides with an average value of 34.4°. This value differs by only 0.2° from the average value derived from the largest validation dataset of 760 crystal structures of bound DNAs with an average occurrence of 262 times of each of the 10 unique dinucleotides. It is noteworthy that HelT is correctly predicted for the CpA, CpG and TpA dinucleotides, for which MD simulations, even with revised force fields (28), report very low HelT values (13).

In addition to performing the validations with experimental data, we used leave-one-out cross-validation to test whether a pentamer is the appropriate size of a sliding window for mining MC data. In each round of cross-validation, we removed one of the 2121 sequences. Then, we recompiled the pentamer query table for the HT method with the remaining MC data and used it to predict the structural feature vectors of the removed sequence. These steps were repeated for each sequence in our training dataset. Structural feature vectors derived from the HT and MC predictions were then concatenated and compared by Spearman’s rank correlation. The resulting correlation coefficients for the respective feature vectors are 0.85 for MGW, 0.92 for Roll, 0.96 for ProT and 0.94 for HelT. These very high correlation coefficients demonstrate that the pentamer model is sufficient to capture the determinants of the MC-predicted DNA shape features.

## CONCLUSIONS

Our previous work and that by others established MGW as an important feature of DNA shape (1,2). However, prior to the development of the DNAshape web server, DNA structural features could not be analysed for large sequence datasets in a HT manner. To embrace the challenges of the genomic era and to be able to infer various DNA structural features that play a role in DNA shape readout, we present a HT approach to derive DNA structural features from massive sequence data. The HT method is based on the assumption that pentanucleotides can be used to describe the sequence–structure degeneracy of the double helix with sufficient accuracy. The DNAshape web server and its underlying HT methodology predict, for the first time, structural features of DNA that are currently established as important elements for protein–DNA recognition (1–3,14,18). The rapid progress in making HT sequencing data available can now realistically be coupled with structural analyses. Providing structural information in a HT manner and at genomic scale will be the necessary basis for a better understanding of protein–DNA binding specificity.

## SUPPLEMENTARY DATA

Supplementary Data are available at NAR Online: Supplementary Tables 1–4, Supplementary Figures 1–8, Supplementary Materials and Methods and Supplementary References [29,30].

## FUNDING

Two Andrew Viterbi Fellowships; USC-Technion Visiting Fellows Program; and American Cancer Society [IRG-58-007-51 to R.R., an Alfred P. Sloan Research Fellow]. Funding for open access charge: USC-Technion Visiting Fellows Program.

*Conflict of interest statement*. None declared.
